# Supplementary material for: Efficacy of preemptive intravenous ibuprofen and dexketoprofen on postoperative opioid consumption in laparoscopic cholecystectomy: Randomized controlled study
Source: PLoS One. 2025 Sep 3;20(9):e0318059. doi: 10.1371/journal.pone.0318059 (PMC12407479; doi:10.1371/journal.pone.0318059)
Supplement: S3 File — (DOCX) [file pone.0318059.s003.docx]

# STUDY PROTOCOL

**Purpose of Research:**

The primary aim of this study is to investigate the effects of preemptive ibuprofen and dexketoprofen use on postoperative opioid consumption in patients undergoing elective laparoscopic cholecystectomy. The secondary aim is to compare the intraoperative hemodynamics, postoperative complications and patient satisfaction of routinely used preemptive drugs ibuprofen and dexketoprofen.

**Scientific Basis and Validity of Medical Research:**

Laparoscopic surgery is the most performed procedure in recent years. The most commonly applied procedure in laparoscopic surgery is laparoscopic cholecystectomy. The main advantages of laparoscopic surgeries are early mobilization and less postoperative pain. The pain observed after laparoscopic cholecystectomy is visceral and can be due to tissue trauma, abdominal distension, chemical irritation of the peritoneum and irritation of the diaphragm by CO2 gas dissolved in the abdomen, and is usually seen in the incision area, shoulder and abdomen (1). In laparoscopic surgery, it causes endocrine and metabolic response together with activation of hypothalamic-pituitary-adrenal axis and sympathetic nervous system. Studies have shown that the severity of trauma is directly proportional to surgical stress. (2) During laparoscopic surgery: tissue trauma, abdominal distension, trauma related to removal of gallbladder cause pain (3). Postoperative pain varies depending on the magnitude of surgical trauma, anesthetic approach, physiological, psychological, emotional and sociocultural factors of the patient (4). Postoperative pain is an acute pain that starts with surgical trauma, gradually decreases and ends with tissue healing. Treatment of acute pain should be rapid and effective. Opioid and non-steroidal anti-inflammatory drugs are widely used for postoperative pain control. (3). Ibuprofen and dexketoprofen NSAIDs Oral forms have been used safely for years. Ibuprofen and dexketoprofen are frequently used in the treatment of mild to moderate pain and in combination with opioids in the treatment of moderate to severe pain (5). The aim of preemptive analgesia is to reduce central sensitization resulting from noxious procedures throughout the perioperative period. Our aim in this study was to investigate the effects of preemptive ibuprofen and dexketoprofen on opioid consumption after laparoscopic cholecystectomy.

**Study Protocol, Methods and Procedures to be Applied:**

90 patients with ASA I-II, ages between 18-65, who underwent laparoscopic cholecystectomy will be included in the study. Patients who started with laparoscopic cholecystectomy and underwent open cholecystectomy, ASA III and IV patients will not be included in our study. All patients will be seen one day in advance and their physical examinations and laboratory findings will be evaluated. All patients included in the study will be informed about VAS one day in advance and will be informed about the pain scoring system numbered 0 to 10 for determining the severity of pain. Patients will be asked to express their pain numerically, with 0 for no pain and 10 for the most severe pain.

The placebo group will be given 100 cc of isotonic solution 30 minutes before preoperatively. The dexketoprofen group will be given 50 mg dexketoprofen in 100 cc via 30 minutes as an infusion. The ibuprofen group will be given 800 mg iv in 100 cc via isotonic solution (all infusions will be completed within 30 minutes). The patients' hemodynamic parameters HR (Heart Rate), SBP (Systolic Blood Pressure), DBP (Diastolic Blood Pressure), MBP (Mean Blood Pressure), SpO2 (Oxygen Saturation) will be recorded every ten minutes during the preoperative and procedure. The duration of surgery will also be recorded. All patients will receive general anesthesia. Anesthesia induction will be performed with 2 mg/kg propofol, 2 mcg/kg fentanyl, and 0.6 mg/kg rocuronium intravenously in all three groups. Anesthesia will be maintained with 8% desflurane, 40% O2, and 1mcg/kg fentanyl. After the surgery, 0.5% bupivacaine will be applied to the trocar entry sites, and 4 cc of infiltration anesthesia will be applied to each trocar entry site by deep and subcutaneous infiltration. At the end of the surgery, 0.015 mg/kg atropine and 0.04 mg/kg neostigmine will be used intravenously to antagonize the muscle relaxant effect. In addition, PCA will be adjusted as a bolus dose of 25 μg fentanyl, a locking time of 10 min, and a maximum of 6 doses per hour without basal infusion. POSTOP ANALGESIA: In addition, PCA will be adjusted as bolus dose 25 μg fentanyl, locking time 10 min, maximum 6 doses per hour without basal infusion.

During laparoscopy, intra-abdominal pressure will be kept at 12-14 mmHg. Patients will be taken to the recovery room. Those with modified Aldrete score 9 and above in the recovery room will be sent to the ward. VAS scores at 1, 2, 4, 6, 12 and 24 hours in the postoperative period will be recorded as VAS rest (lying down). The nurse/doctor who questions the patients' VAS scores will be unaware of the drugs used for analgesia and the grouping. Patients with VAS scores ≥ 4 will be administered 50 mg tramadolol 100 cc mai as an additional IV. Application times will be recorded. All patients will be monitored for nausea and vomiting, dry mouth, itching, palpitations and headaches that occur in the first 24 hours. At the end of the study, patients will be questioned about their satisfaction with post-operative pain or other discomfort. They will be asked to evaluate their satisfaction as poor=1, moderate=2, good=3, and the results will be recorded, and the study will be concluded.

**Statistical Data Analysis:**

According to our previous preliminary study, the standard deviation for postoperative opioid consumption was found to be 8. The Z table value was taken as 1.96 at 5% Type I error and 95% confidence limit. According to the maximum value of 20% of the Type II error predicted for 5% Type I error, the effect size was taken as 3 with 80% power value. Accordingly, the sample size was found as 27 using the equation n = Z2xσ2/d2. However, the sample size was taken as 30 in the study. Descriptive statistics for continuous variables among the features emphasized are expressed as Mean, Standard Deviation, Minimum and Maximum values, while for categorical variables they will be expressed as number and percentage. In terms of continuous variables, One-Way Analysis of Variance or Kruskal-Wallis analysis will be used to compare groups. Following the variance analysis, Duncan multiple comparison test will be used to determine different groups. In addition, Repeated Measurement Analysis of Variance or Friedman test will be used to determine whether there is a difference between the measured regions. In determining the relationship between variables, Pearson correlation coefficients will be calculated separately for each group. In comparing groups in terms of categorical variables, a ratio comparison will be made. In the calculations, the statistical significance level will be taken as 5% and SPSS (ver:23) statistical package program will be used for the calculations.

**References**

1**.** Lee IO, Kim SH, Kong MH, et al. Pain after lapa­roscopic cholecystectomy: the effect and timing of incisional and intraperitoneal bupivacaine. Can J Anaesth 2001;48:545-50.

2. Kalaycı MU, Akın BV, Alış H, Kapan S, Turhan AN, Yiğitbaş H, Hatipoğlu S, Aygün E. Laparoskopik kolesistektomide meydana gelen iyatrojenik safra kesesi perforasyonlarının erken postoperatif dönemdeki ağrı derinliğine ve hastanede kalış süresine etkisi. Bakırköy Tıp Dergisi. 2006; 2:55-58.

3. Memedov C, Menteş Ö, Şimşek A, Keçe C, Yağcı G, Harlak A, Coşar A, Tufan T. Laparoskopik kolesistektomi sonrası postoperatif ağrının önlenmesinde çoklu bölgeye lokal anestezik infiltrasyonu: Ropivakain ve prilokainin plasebo kontrollü karşılaştırılması. Gülhane Tıp Dergisi. 2008; 50:84-90.

4.Bayar M, İlhan Y, Önal A, Akkuş M, Çifter Ç. Laparoskopik kolesistektomilerde intraperitoneal bupivakain uygulamasının postoperatif ağrı ve katekolamin düzeylerine etkileri. Ağrı Dergisi. 1998; 10 :30-34.

5.Moss JR, Watcha MF, Bendel LP, McCarthy DL, Witham SL, Glover CD. A multicenter, randomized, double-blind placebo-controlled, single dose trial of the safety and efficacy of intravenous ibuprofen for treatment of pain in pediatric patients undergoing tonsillectomy. Paediatr Anaesth. 2014 May;24(5):483-9. doi: 10.1111/pan.12381. Epub 2014 Mar 20
